# A dynamic visualization clinical tool constructed and validated based on the SEER database for screening the optimal surgical candidates for bone metastasis in primary kidney cancer

**DOI:** 10.1038/s41598-024-54085-x

**Published:** 2024-02-12

**Authors:** Liming Jiang, Yuexin Tong, Jun Wang, Jiajia Jiang, Yan Gong, Dejin Zhu, Linyang Zheng, Dongxu Zhao

**Affiliations:** 1 Department of Orthopedics, The China-Japan Union Hospital of Jilin University, No. 126 Xiantai Street, Changchun, 130033 Jilin People’s Republic of China; 2 Department of Orthopedics, Rizhao People’s Hospital, Rizhao, 276800 Shandong People’s Republic of China

**Keywords:** Kidney cancer, Bone metastasis, Optimal surgical candidates, Primary tumor resection, SEER database, Bone cancer, Cancer epidemiology, Cancer models, Cancer screening, Metastasis, Urological cancer, Diseases, Medical research, Nephrology, Oncology, Risk factors, Urology

## Abstract

The implementation of primary tumor resection (PTR) in the treatment of kidney cancer patients (KC) with bone metastases (BM) has been controversial. This study aims to construct the first tool that can accurately predict the likelihood of PTR benefit in KC patients with BM (KCBM) and select the optimal surgical candidates. This study acquired data on all patients diagnosed with KCBM during 2010–2015 from the Surveillance, Epidemiology, and End Results (SEER) database. Propensity score matching (PSM) was utilized to achieve balanced matching of PTR and non-PTR groups to eliminate selection bias and confounding factors. The median overall survival (OS) of the non-PTR group was used as the threshold to categorize the PTR group into PTR-beneficial and PTR-Nonbeneficial subgroups. Kaplan–Meier (K–M) survival analysis was used for comparison of survival differences and median OS between groups. Risk factors associated with PTR-beneficial were identified using univariate and multivariate logistic regression analyses. Receiver operating characteristic (ROC), area under the curve (AUC), calibration curves, and decision curve analysis (DCA) were used to validate the predictive performance and clinical utility of the nomogram. Ultimately, 1963 KCBM patients meeting screening criteria were recruited. Of these, 962 patients received PTR and the remaining 1061 patients did not receive PTR. After 1:1 PSM, there were 308 patients in both PTR and non-PTR groups. The K–M survival analysis results showed noteworthy survival disparities between PTR and non-PTR groups, both before and after PSM (p < 0.001). In the logistic regression results of the PTR group, histological type, T/N stage and lung metastasis were shown to be independent risk factors associated with PTR-beneficial. The web-based nomogram allows clinicians to enter risk variables directly and quickly obtain PTR beneficial probabilities. The validation results showed the excellent predictive performance and clinical utility of the nomograms for accurate screening of optimal surgical candidates for KCBM. This study constructed an easy-to-use nomogram based on conventional clinicopathologic variables to accurately select the optimal surgical candidates for KCBM patients.

## Introduction

Kidney cancer (KC) is a prevalent urologic malignancy with a rising incidence rate^1^. Throughout 2023, an estimated 81,800 newly diagnosed cases of KC will arise in the United States with 14,890 of these patients facing the threat of mortality^2^. Additionally, at the time of initial diagnosis, almost one-third of new KC patients exhibit metastases to other sites^3^. Bone is the second most prevalent site of KC metastasis, comprising 29.5% of cases^4^. Since the use of biologic and targeted antiangiogenic therapies in KC patients and the resulting increase in overall survival, bone metastases have become even more prevalent^3,5^. In addition, bone may be a new shelter for targeted therapies in metastatic KC^6^. Compared to other cancers, BM in KC is more destructive and has a higher incidence of skeletal-related events (SRE)^7^. Over 71% of KC patients with BM (KCBM) may develop osteolytic lesions, leading to numerous serious SRE^8^. The most frequent SREs include pain, anemia, pathological fractures, and spinal cord compression^6^. The common sites of BM are centered on the axial bones (pelvis, spine, and ribs) with fewer sites in the extremities^9^. More frustratingly, the prognosis for KC patients who develop BM is abysmal, with a median survival of only 10–12 months^8,10^. Therefore, it is becoming increasingly important to explore ways to improve management modalities and improve survival benefits in KCBM patients.

In recent years, multi-agent combination chemotherapy regimens have become increasingly prevalent in the treatment of metastatic KC^11,12^. In accordance with current clinical management guidelines, metastatic KC patients who undergo primary tumor resection (PTR) in conjunction with chemotherapy exhibit an increased overall response rate and experience longer progression-free survival, but none of them exceeded the fixed noninferiority limit^13^. The therapeutic benefits of radiotherapy in KC are reported to be limited. Radiotherapy is not only unsuitable for early-stage KC, but even in advanced stages, it should be used with caution after comprehensive evaluation^14^. Furthermore, the optimal dose of radiotherapy remains unclear^15^.

Surgical resection could improve the prognosis of early-stage KC patients, which has been confirmed in many studies^16,17^. However, while many studies have shown that surgical resection could improve the survival of patients with advanced KC, the trauma of surgery and the impact of postoperative complications on survival have been widely scrutinized^18–20^. In addition, studies by Silberstein et al. have shown that radical nephrectomy for metastatic KC is a more demanding procedure and more prone to complications than for localized KC^21^. Patients recommended for PTR tend to be characterized by few risk factors and fitness status^22^. In a retrospective study, Zekri et al. found that PTR combined with systemic therapy could prolong survival in KCBM^23^. Moreover, in a study investigating the prognostic factors of BM from KC, Wang et al. pointed out that resection of the primary site tumor was a beneficial independent prognostic factor^24^.

Even though some studies have reported that radiotherapy and chemotherapy are not particularly effective in KCBM, local recurrence of the tumor and more extensive distant metastases make PTR still subject to many limitations^25–27^. Building on previous research, we formulated the hypothesis that not all patients with KCBM would reap the benefits of PTR. How to differentiate between PTR-beneficial and PTR-Nonbeneficial patients, as well as to construct accurate nomogram for predicting the probability of PTR-benefits are urgently needed at this time. This study aims to accurately screen the most suitable PTR candidates for KCBM, quantify and illustrate the probability of PTR-benefits, and provide individualized management programs that will ultimately improve survival.

## Methods

### Study patient

The Surveillance, Epidemiology, and End Results (SEER) database is the largest publicly accessible cancer database, covering 18 cancer registries and approximately 28% of the US population^28^. In this study, the data used to construct and internally validate the nomogram were obtained from the SEER database (SEER*Stat 8.4.1, username: 15685 Nov-2020). The SEER database began recording details of cancer metastases after 2010, so we recruited all KCBM patients between 2010–2015 as the initial cohort for this study. The inclusion criteria were: (1) the time of first diagnosis was between 2010–2015; (2) KC confirmed by site recode ICD-O-3/WHO 2008 (kidney and renal pelvis); (3) confirmed by positive histology; (4) KC was the first primary tumor; (5) BM were confirmed together with the first diagnosis of KC. Exclusion criteria: (1) unknown information on study variables (race, T stage, N stage, tumor size, surgery, brain/liver/lung metastasis, insurance, and marital status); (2) survival time less than 1 month; (3) incomplete follow-up.

In addition, we recruited 53 KCBM patients who underwent PTR at the China-Japan Union Hospital of Jilin University between 2010 and 2017 as an external validation set. Detailed information on demographic variables was obtained from the medical record system and follow-up records. Clinicopathologic characteristics were evaluated by two pathologists under double-blind conditions. Inclusion and exclusion criteria for the external validation set were strictly identical to those described above. Since neither the SEER database nor the external validation set contained identifiable individual information and this was a retrospective cohort study, the ethical review committee exempted ethical review and informed consent.

The variables of this study included demographic information (age, sex, race, insurance, marital status), clinicopathologic characteristics (histological type, laterality, tumor size, grade, T/N stage, brain/liver/lung metastasis), and treatment details (surgery, chemotherapy, radiotherapy). Age was categorized as < 50, 50–70, and > 70 years, and tumor size was categorized as < 40, 40–80, and > 80 mm^8,29^. Surgery represents primary tumor resection (PTR), not metastatic tumor resection. Overall survival (OS) was defined as the time from initial cancer diagnosis to death from any cause. Cancer-specific survival^30^ was defined as the time from diagnosis to death from cancer-related causes only. The hypothesis of this study is that patients in the PTR group, whose OS is greater than the median OS of the non-PTR group, would benefit from surgery.

### Propensity score matching analysis

The KCBM patients cohort was divided into PTR group and non-PTR group according to whether they underwent PTR or not after screening by inclusion and exclusion criteria. To reduce the effect of selection bias and confounders on the study cohort, we used propensity score matching (PSM) for the PTR group and non-PTR group in a 1:1 match (caliper: 0.05). We calculated the standardized mean difference of patients' baseline variables in the two groups before and after matching, and performed Chi-squared test and Fisher's exact test to validate the differences between baseline variables to demonstrate the results of propensity score matching.

### Prediction nomogram construction and validation

Kaplan–Meier (KM) survival analysis and log-rank test were performed on the non-PTR group after PSM, and the cumulative 50% survival rate corresponded to the survival time, which was the median survival time. Then, the PTR group was divided by the median overall survival of the non-PTR group. In the PTR group, patients whose overall survival was greater than the median overall survival of the non-PTR group were considered to benefit from PTR. Conversely, they were not considered to benefit from PTR. The PTR group after PSM was randomized into a training set (70%) and an internal validation set (30%). Univariate and multivariate logistic regression was applied to the training set to identify independent risk factors associated with PTR benefit. Based on the independent risk factors, nomograms (static and web-based versions) were constructed in R software.

Internal and external validation sets were used to validate the predictive accuracy and clinical utility of the nomogram. receiver operating characteristic curve (ROC) and area under the curve (AUC) were used to validate the predictive efficacy of the nomogram. The values of AUC varied from 0.5 to 1.0, with values greater than 0.75 indicating that the nomogram had excellent predictive accuracy^31^. Calibration curves were used to verify the consistency between the predicted values of the nomogram and actual clinical observations. The closer the dotted line representing the predicted values is to the solid line representing the actual values, the better the predictive efficacy of the predictive tool^32^. Decision curve analyses (DCA) are a very useful way of assessing the net clinical benefits of a nomogram^33^. If the nomogram provides net clinical benefits that are much greater than “all therapy” and “no treatment,” this suggests the nomogram has excellent clinical utility.

### Statistical analysis

The statistical analyses in this study were all performed in SPSS (version 27.0) and R software (version 4.2.2, https://www.r-project.org/). Statistical analyses performed in SPSS included: (1) Univariate and multivariate Cox regression analyses to verify whether PTR was an independent prognostic factor for the pre-PSM KCBM patients; (2) Differences between baseline variables in the pre- and post-PSM sets (Chi-squared test and Fisher’s exact test); (3) In the post-PSM PTR group, univariate and multivariate logistic regressions were performed to analyze the independent risk factors on PTR-beneficial correlations. All other statistical analyses were performed in R software. The R packages used for K–M survival analyses were “survival” and “survminer”. The R package used for PSM was “MatchIt”. The R packages used to construct static and dynamic nomograms were “rms” and “DynNom”. The R packages used for calibration curves are “rms” and “foreign”. DCA was performed with the function “stdca.R”. All statistical tests with a p-value < 0.05 [95% confidence interval (CI)] were considered statistically significant^34^.

## Results

### Clinicopathologic characteristics of patients before and after PSM

**Figure 1:**
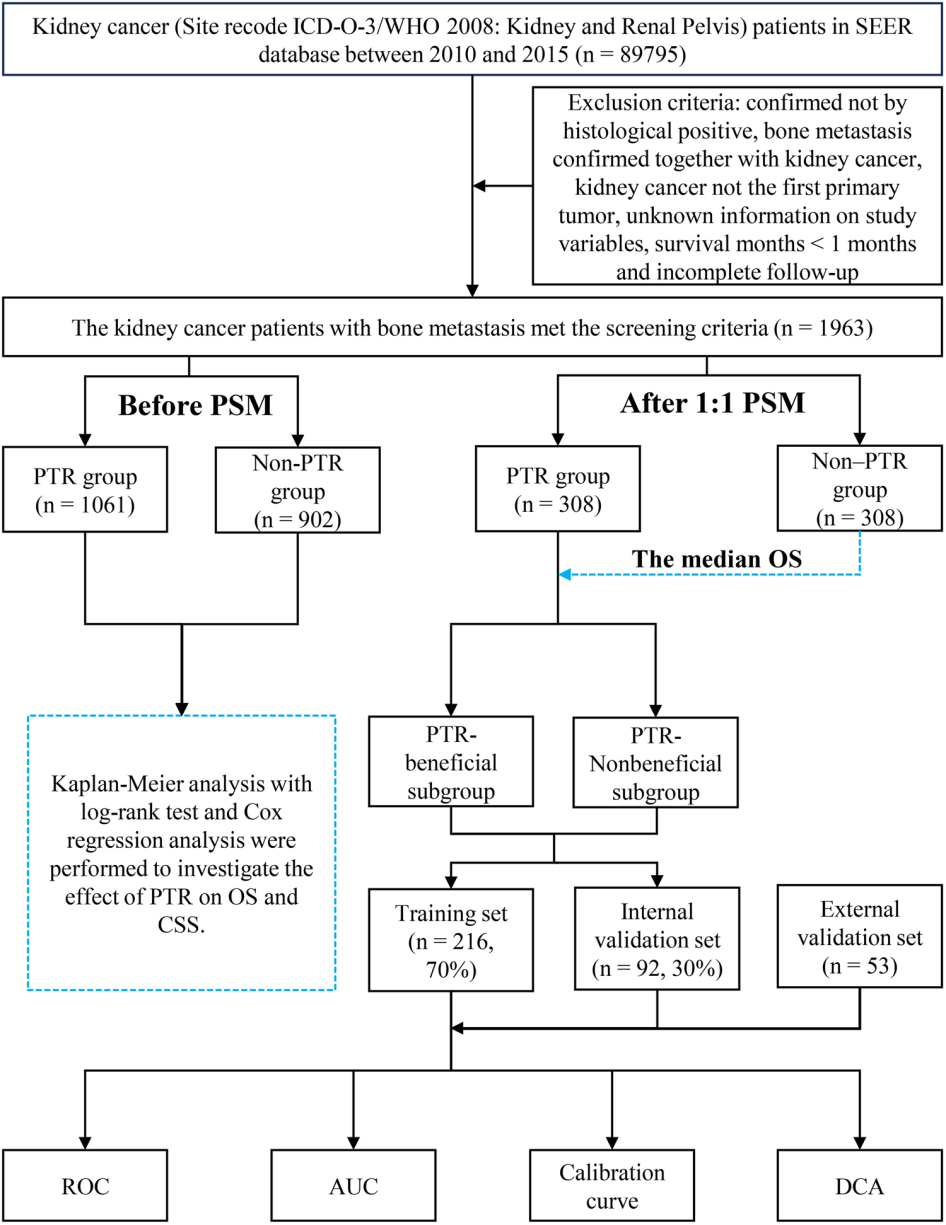
Patients’ selection and workflow of this study. *SEER* The Surveillance, Epidemiology, and End Results, *PSM* propensity score matching, *PTR* primary tumor resection, *OS* overall survival, *CSS* cancer specific survival, *ROC* receiver operating characteristic curve, *AUC* area under the curve, *DCA* decision curve analyses.

A total of 1963 KCBM patients met screening criteria in the SEER database between 2010–2015. Among them, 902 patients (46%) received PTR and 1061 patients (54%) did not receive PTR. However, there were significant differences in the baseline variables compared between the two groups of KCBM patients. The variables of age, race, histologic type, laterality, tumor size, grade, T/N stage, brain/liver/lung metastasis, chemotherapy, radiotherapy, and marital status had a p-value of < 0.05 (Table 1). After 1:1 PSM, the differences in all baseline variables between the PTR (n = 308, 50%) and PTR (n = 308, 50%) groups were balanced (all p > 0.05 and all SMD < 0.1) (Table 1 and Supplementary Fig. 1). Next, taking the median OS of 6 months in the non-PTR group as a threshold, the PTR group was subdivided into PTR beneficial (OS > 6 months) and PTR non-beneficial (OS ≤ 6 months). Finally, patients in the PTR group were randomized in a 7:3 ratio into a training set (n = 216, 70%) and a validation set (n = 92, 30%). In addition, 53 KCBM patients who underwent PTR from the China-Japan Union Hospital of Jilin University were recruited as an external validation set to further validate the predictive performance of the nomogram. Figure 1 illustrates the data screening and workflow of this study (Fig. 1).

**Table 1:** Clinical and pathological characteristics for kidney cancer patients with bone metastasis before and after PSM.

Variables	Before PSM		P-value	After PSM		P-value
	PTR group N = 1061, %	Non-PTR group N = 902, %		PTR group N = 308, %	Non-PTR group N = 308, %	
Age			< 0.001			0.914
< 50	91 (8.58)	135 (14.97)		33 (10.71)	35 (11.36)	
50–70	658 (62.02)	604 (66.96)		201 (65.26)	203 (65.91)	
> 70	312 (29.41)	163 (18.07)		74 (24.03)	70 (22.73)	
Sex			0.463			0.657
Female	329 (31.01)	265 (29.38)		86 (27.92)	92 (29.87)	
Male	732 (68.99)	637 (70.62)		222 (72.08)	216 (70.13)	
Race			0.096			0.845
Black	118 (11.12)	75 (8.31)		26 (8.44)	25 (8.12)	
Other	67 (6.31)	65 (7.21)		13 (4.22)	16 (5.19)	
White	876 (82.56)	762 (84.48)		269 (87.34)	267 (86.69)	
Histological type			< 0.001			0.800
8310/3	417 (39.30)	530 (58.76)		160 (51.95)	154 (50.00)	
8312/3	428 (40.34)	132 (14.63)		72 (23.38)	79 (25.65)	
Other	216 (20.36)	240 (26.61)		76 (24.68)	75 (24.35)	
Laterality			0.003			0.629
Left	524 (49.39)	506 (56.10)		149 (48.38)	156 (50.65)	
Right	537 (50.61)	396 (43.90)		159 (51.62)	152 (49.35)	
Tumor size			< 0.001			0.970
< 40 mm	159 (14.99)	73 (8.09)		37 (12.01)	38 (12.34)	
40–80 mm	482 (45.43)	404 (44.79)		152 (49.35)	149 (48.38)	
> 80 mm	420 (39.59)	425 (47.12)		119 (38.64)	121 (39.29)	
Grade			< 0.001			0.842
I–II	75 (7.07)	139 (15.41)		48 (15.58)	51 (16.56)	
III–IV	142 (13.38)	626 (69.40)		132 (42.86)	125 (40.58)	
Unknown	844 (79.55)	137 (15.19)		128 (41.56)	132 (42.86)	
T stage			< 0.001			0.835
T1	425 (40.06)	168 (18.63)		94 (30.52)	97 (31.49)	
T2	283 (26.67)	129 (14.30)		60 (19.48)	57 (18.51)	
T3	211 (19.89)	510 (56.54)		109 (35.39)	102 (33.12)	
T4	142 (13.38)	95 (10.53)		45 (14.61)	52 (16.88)	
N stage			< 0.001			1.000
N0	641 (60.41)	629 (69.73)		197 (63.96)	197 (63.96)	
N1	420 (39.59)	273 (30.27)		111 (36.04)	111 (36.04)	
Brain metastasis			< 0.001			0.509
No	907 (85.49)	838 (92.90)		273 (88.64)	279 (90.58)	
Yes	154 (14.51)	64 (7.10)		35 (11.36)	29 (9.42)	
Liver metastasis			< 0.001			0.661
No	827 (77.95)	808 (89.58)		261 (84.74)	256 (83.12)	
Yes	234 (22.05)	94 (10.42)		47 (15.26)	52 (16.88)	
Lung metastasis			< 0.001			0.375
No	508 (47.88)	544 (60.31)		157 (50.97)	169 (54.87)	
Yes	553 (52.12)	358 (39.69)		151 (49.03)	139 (45.13)	
Radiotherapy			0.015			0.806
No	412 (38.83)	400 (44.35)		127 (41.23)	131 (42.53)	
Yes	649 (61.17)	502 (55.65)		181 (58.77)	177 (57.47)	
Chemotherapy			0.001			0.399
No	346 (32.61)	361 (40.02)		114 (37.01)	103 (33.44)	
Yes	715 (67.39)	541 (59.98)		194 (62.99)	205 (66.56)	
Insurance			0.965			1.000
No	35 (3.30)	31 (3.44)		13 (4.22)	12 (3.90)	
Yes	1026 (96.70)	871 (96.56)		295 (95.78)	296 (96.10)	
Marital status			< 0.001			0.868
No	458 (43.17)	296 (32.82)		113 (36.69)	116 (37.66)	
Yes	603 (56.83)	606 (67.18)		195 (63.31)	192 (62.34)	

*PSM* propensity score matching, *PTR* primary tumor resection.

### Correlation between primary tumor resection and survival in KCBM patients

**Figure 2:**
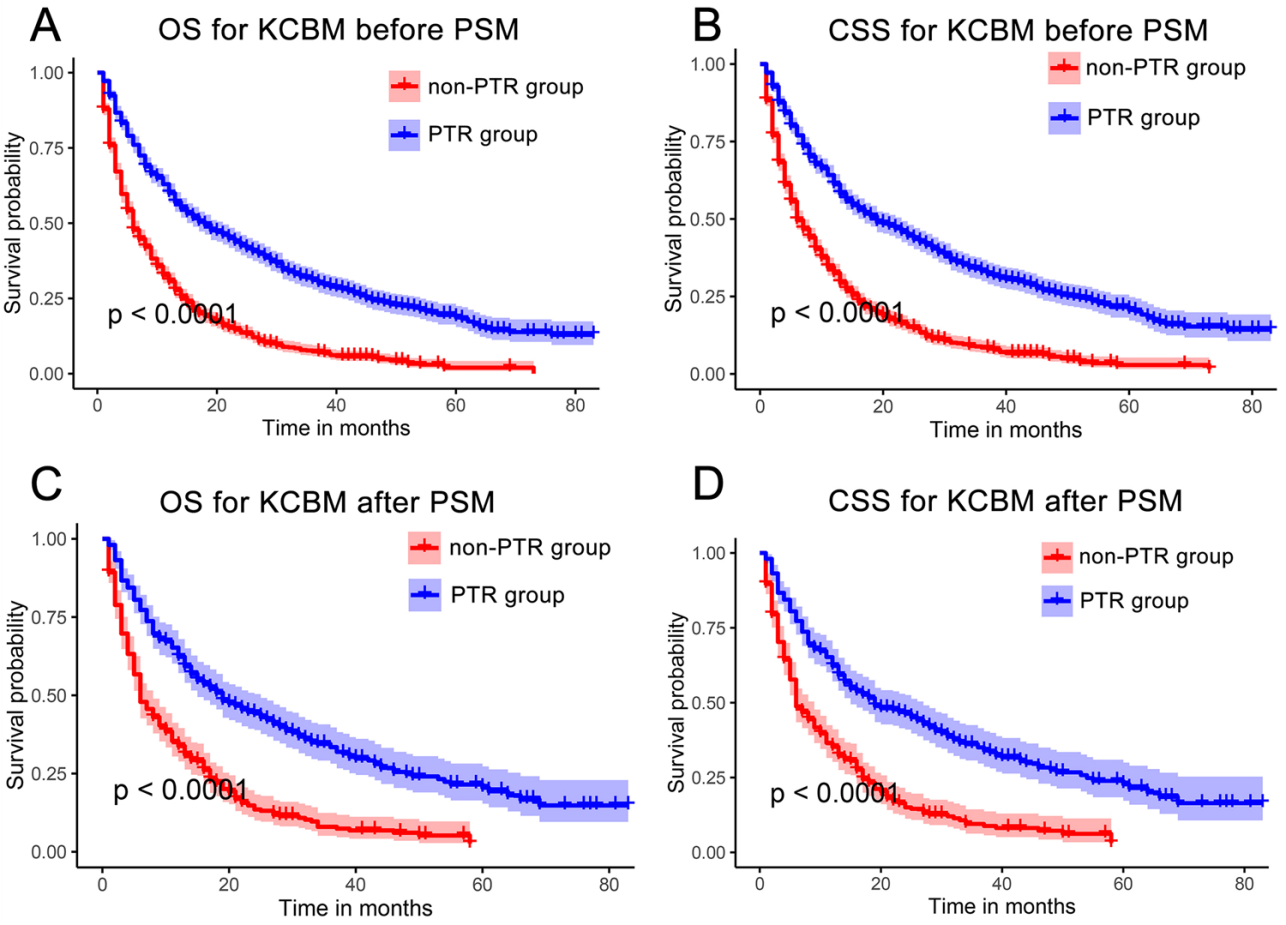
The impact of primary tumor resection on the survival outcomes of kidney cancer patients with bone metastasis. Kaplan–Meier survival curves of OS before PSM (**A**) and after PSM (**B**) and of CSS before PSM (**C**) and after PSM (**D**) in the PTR and non-PTR groups. *OS* overall survival, *CSS* cancer specific survival, *PSM* propensity score matching, *PTR* primary tumor resection.

**Figure 3:**
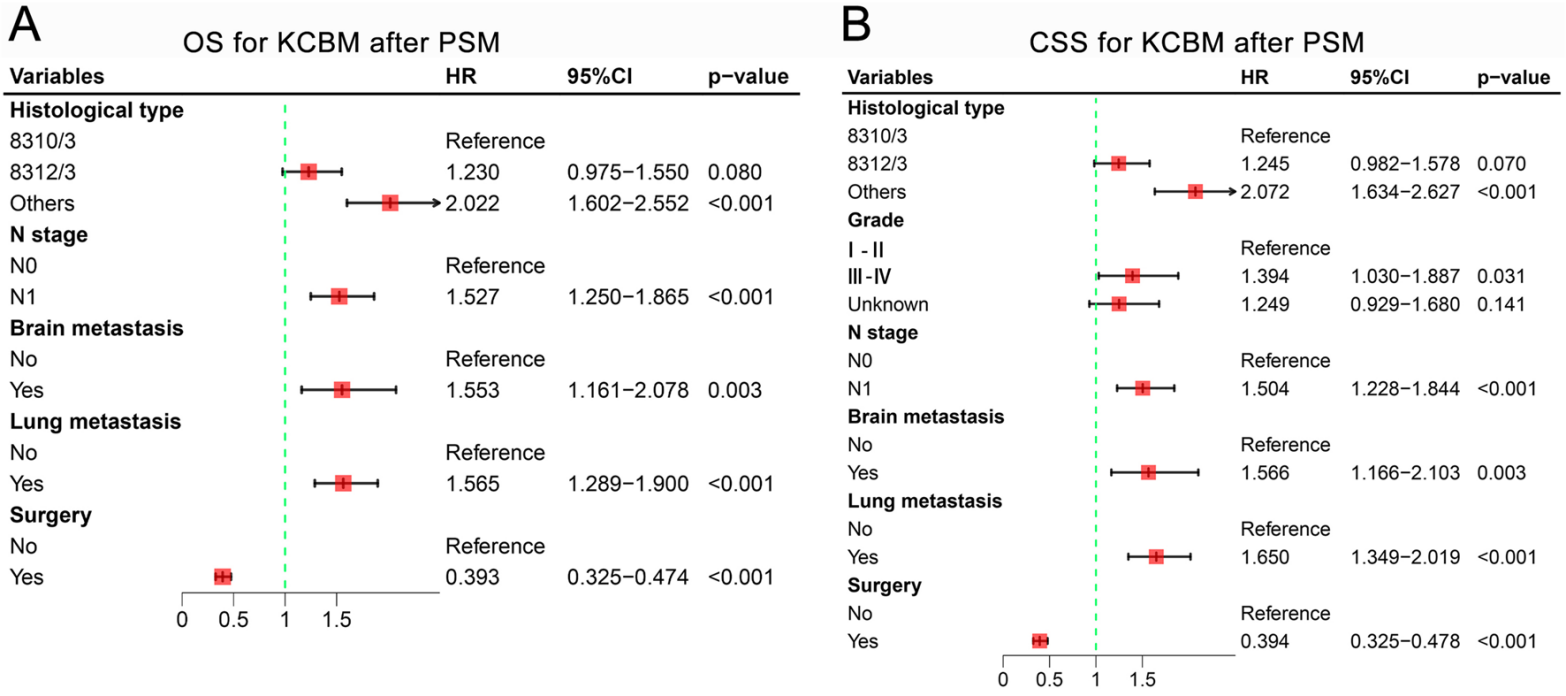
The forest plots for illustrating the results of multivariate Cox regression analysis of OS after PSM (**A**) and CSS after PSM (**B**) in KCBM patients. *KCBM* kidney cancer patients with bone metastasis, *HR* hazard ratio, *OS* overall survival, *CSS* cancer specific survival, *PSM* propensity score matching.

In the results of K–M survival analysis, the OS and CSS of the PTR group were superior to those of the non-PTR group both before and after PSM, and the differences were statistically significant (p < 0.001) (Fig. 2A–D). In addition, the median OS (18.0 vs. 6.0 months, p < 0.001) and CSS (19.0 vs. 6.0 months, p < 0.001) of the PTR group were also superior to those of the non-PTR group before PSM. Consistent results were also seen after PSM, with the median OS (19.0 vs. 6.0 months, p < 0.001) and CSS (19.0 vs. 6.0 months, p < 0.001) of the PTR group being superior to that of the non-PTR group. In addition, the results of multivariate Cox regression analyses showed that primary site tumor resection was an independent prognostic factor for KCBM patients both before and after PSM (Supplementary Fig. 2 and Fig. 3).

### Nomogram construction and assessment performance validation

**Figure 4:**
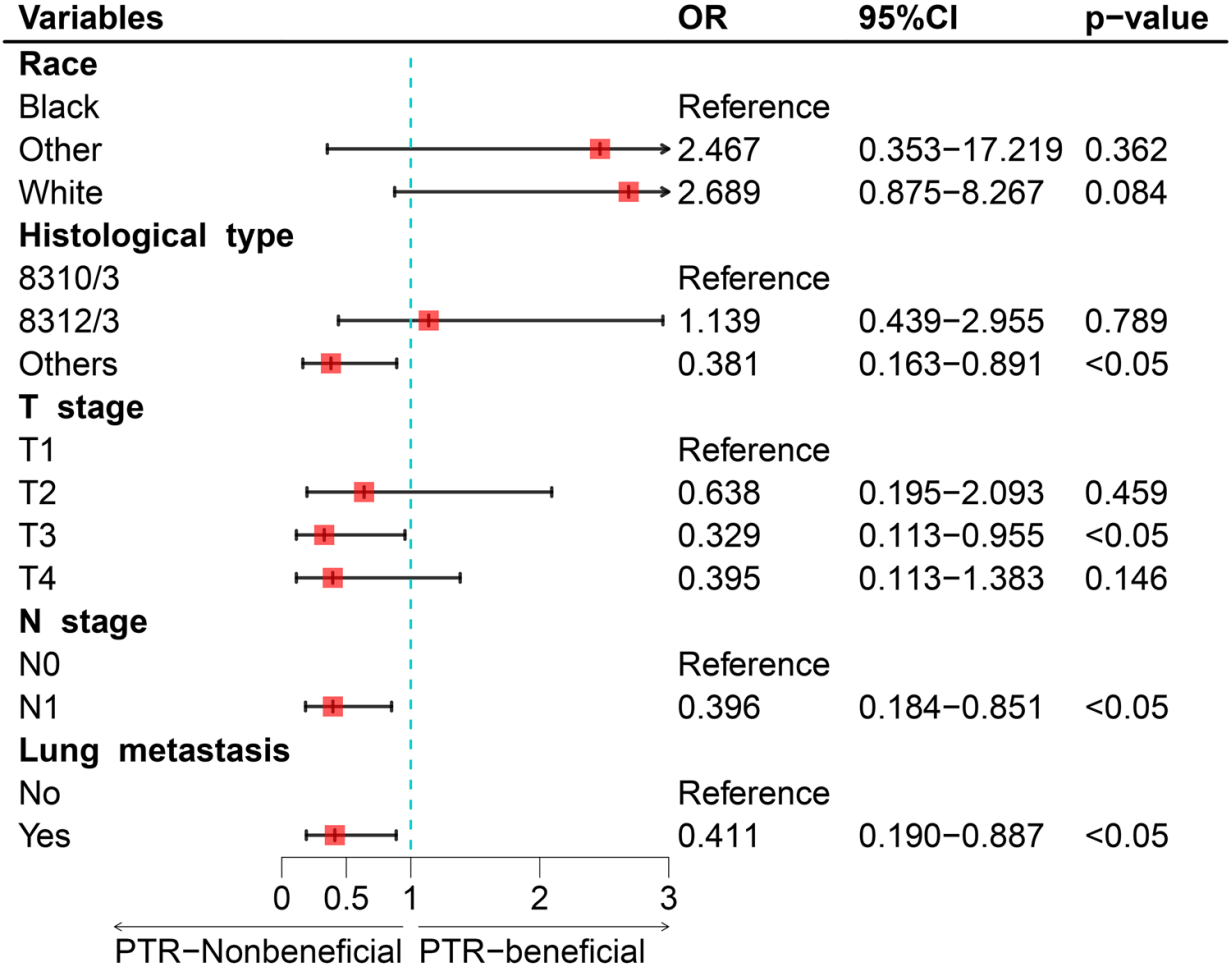
The forest plots for illustrating the results of multivariate logistic regression analyses of independent risk factors associated with PTR benefit in kidney cancer patients with bone metastasis. *PTR* primary tumor resection, *OR* odds ratio.

**Figure 5:**
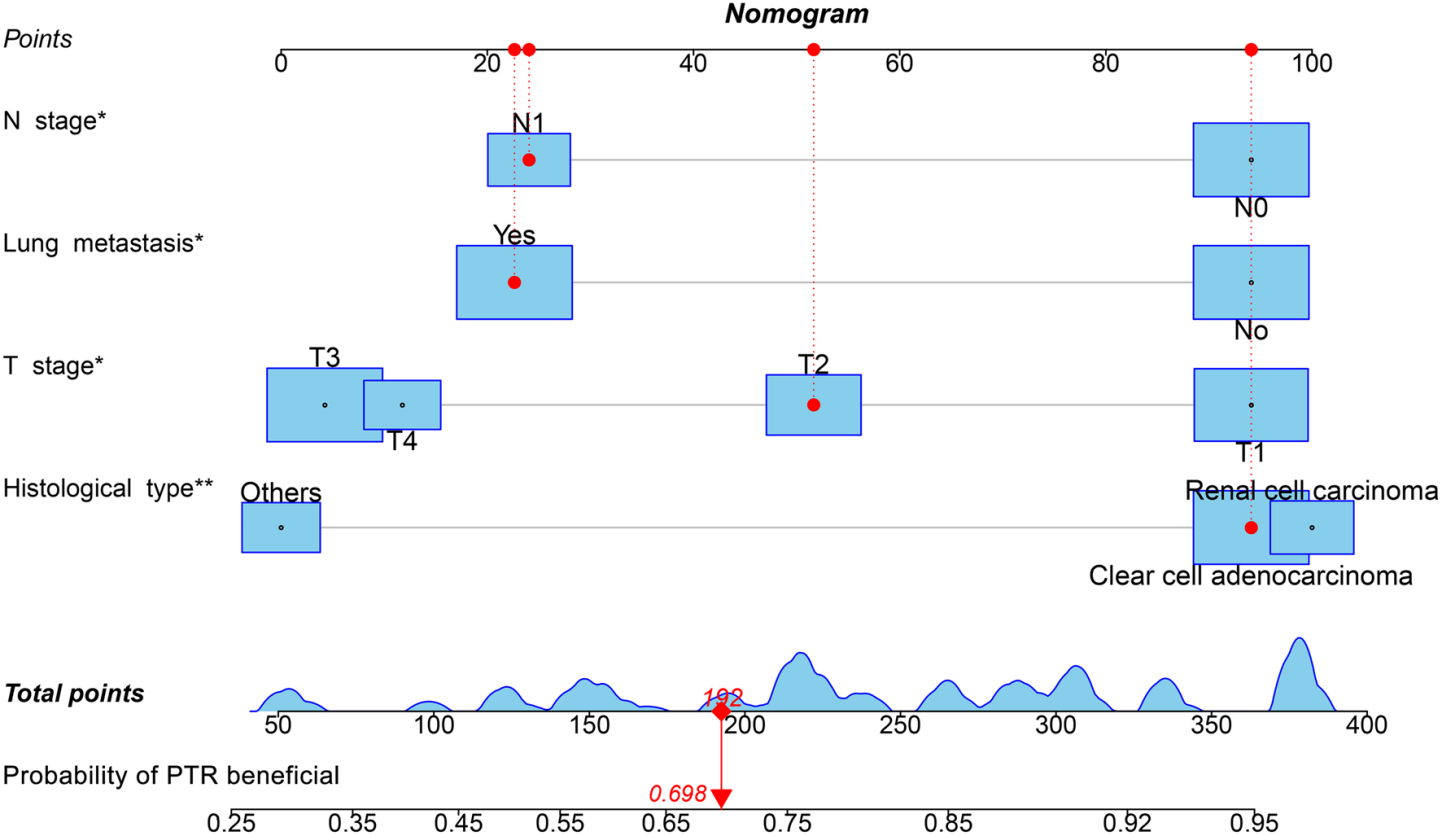
The nomogram to predict the probability of PTR benefit in kidney cancer patients with bone metastasis. *PTR* primary tumor resection.

**Figure 6:**
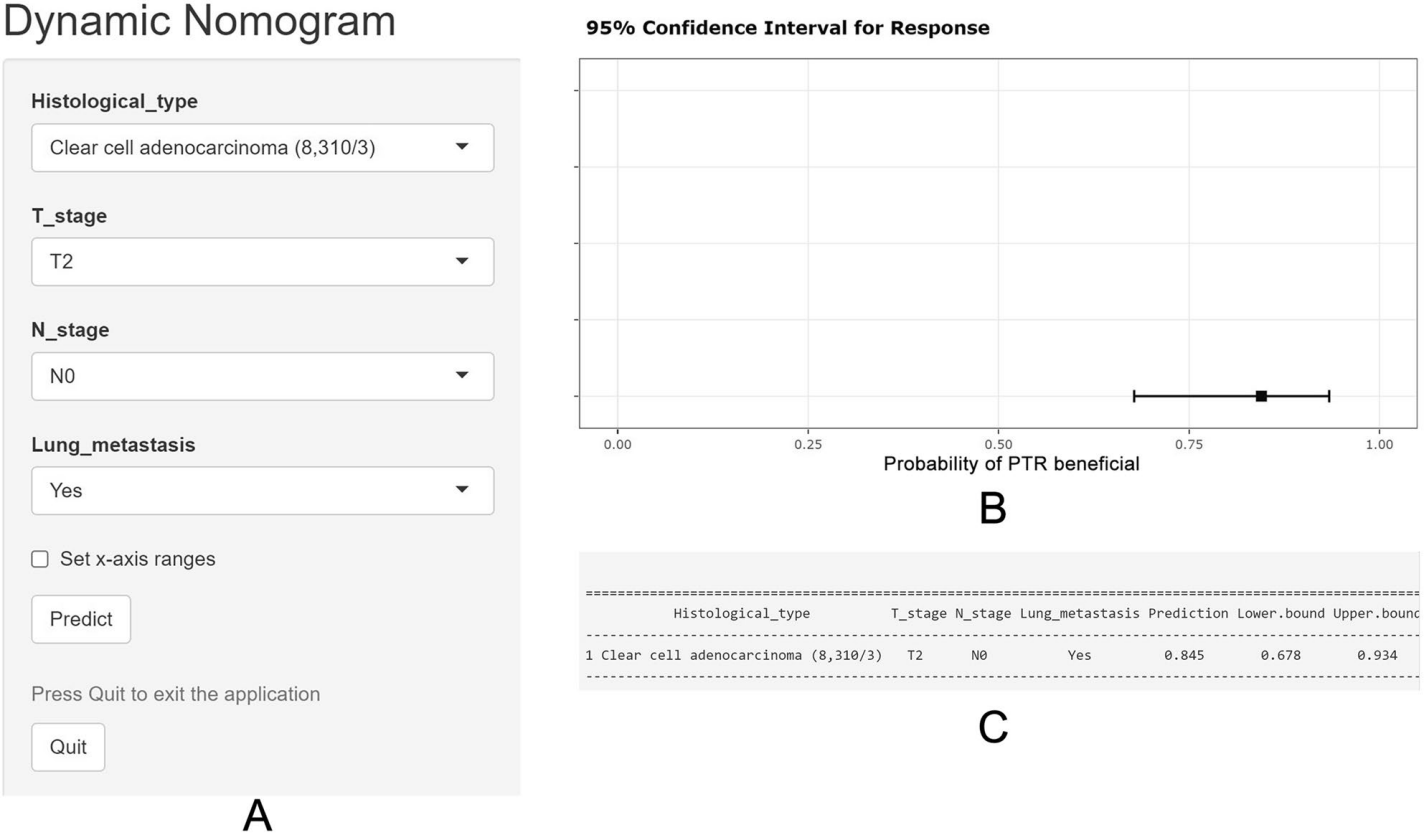
The web-based nomogram to predict the probability of PTR benefit in kidney cancer patients with bone metastasis. (**A**) The operational boxes for entering independent risk variables and predicting the PTR benefit probability. (**B**) 95% confidence intervals of the PTR probabilities for this patient. (**C**) Numerical summary of the PTR probabilities for this patient. Due to a large number of visitors to the webpage, if the application cannot be used normally, please click “Quilt” or “Reload” in the lower-left corner to try again. *PTR* primary tumor resection.

**Figure 7:**
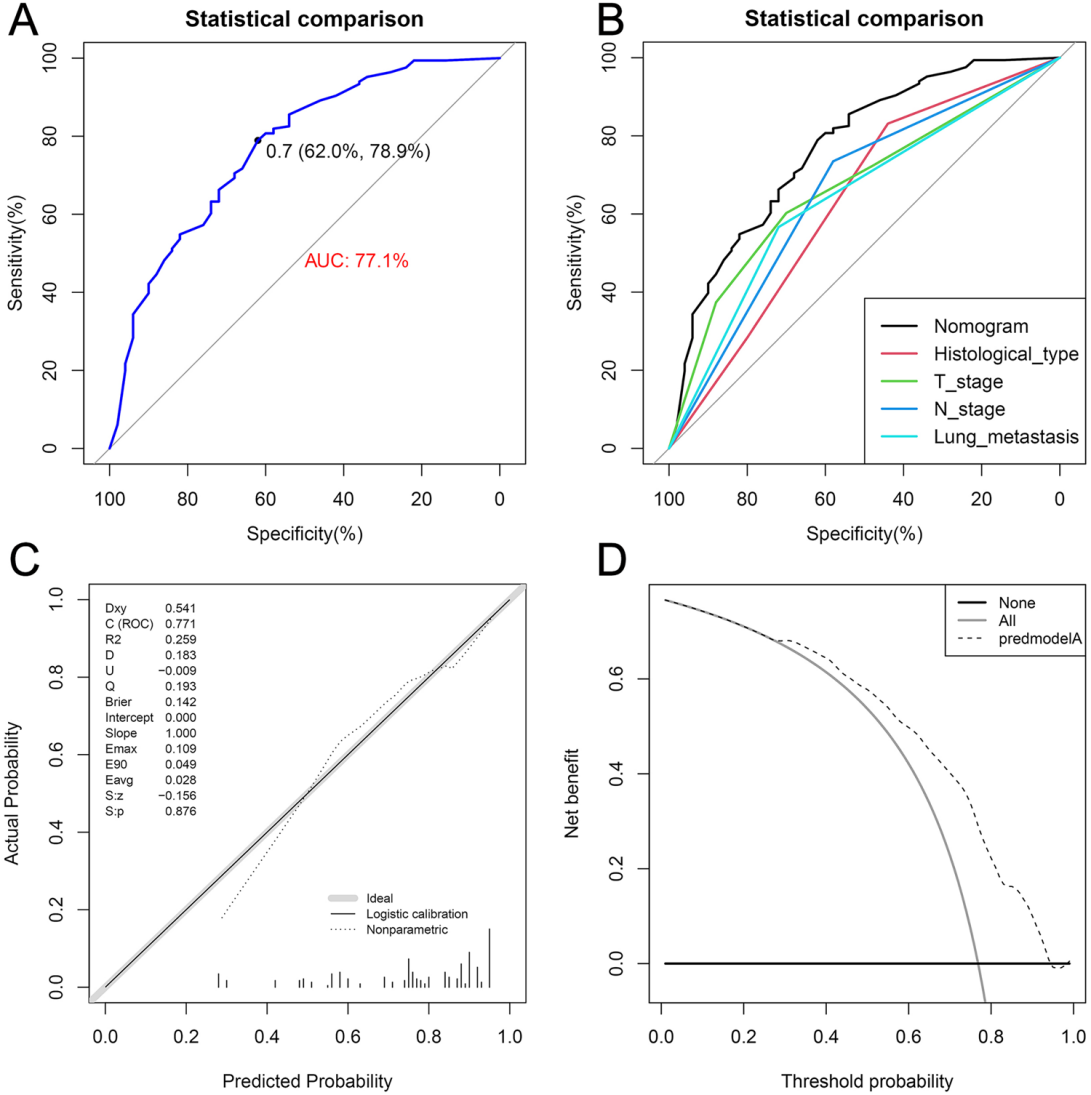
The ROC curve (**A**), comparison of the value of AUC (**B**), calibration curve (**C**) and DCA curve (**D**) of the training set. *ROC* receiver operating characteristic curve, *AUC* area under the curve, *DCA* decision curve analyses.

**Figure 8:**
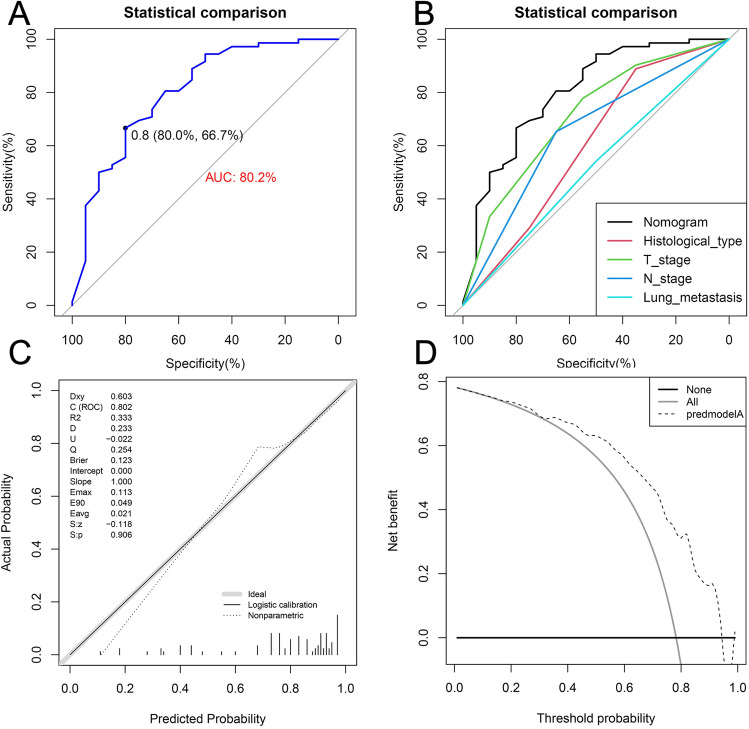
The ROC curve (**A**), comparison of the value of AUC (**B**), calibration curve (**C**) and DCA curve (**D**) of the internal validation set. *ROC* receiver operating characteristic curve, *AUC* area under the curve, *DCA* decision curve analyses.

The results of univariate and multivariate logistic regression analyses of the training set indicated that histologic type, T/N stage, and lung metastasis were independent risk factors associated with PTR benefit (Table 2 and Fig. 4). Based on these independent risk factors, the static and web-based nomograms were constructed to predict the probability of KCBM patients benefiting from PTR (Figs. 5 and 6, https://kcbmptrbeneficial.shinyapps.io/DynNomapp/). The newly constructed nomogram produced ROCs in the training and internal validation set demonstrated that the nomogram has excellent overall predictive efficacy, the AUC was 0.771 (95% CI 0.694–0.847) in the training set and 0.802 (95% CI 0.684–0.919) in the internal validation set (Figs. 7A and 8A).

**Table 2:** Univariate and multivariate logistic analyses of factors related to primary tumor resection in kidney cancer patients with bone metastasis.

Variables	Univariate analysis			Multivariate analysis		
	OR	95%CI	P-value	OR	95%CI	P-value
Age						
< 50	Reference					
50–70	0.675	0.185–2.463	0.552			
> 70	0.373	0.097–1.436	0.152			
Sex						
Female	Reference					
Male	1.396	0.691–2.820	0.353			
Race						
Black	Reference			Reference		
Other	2.864	0.473–17.351	0.252	2.467	0.353–17.219	0.362
White	3.105	1.202–8.020	0.019	2.689	0.875–8.267	0.084
Histological type						
8310/3	Reference			Reference		
8312/3	0.930	0.398–2.174	0.866	1.139	0.439–2.955	0.789
Other	0.252	0.119–0.535	< 0.001	0.381	0.163–0.891	0.026
Laterality						
Left	Reference					
Right	0.786	0.416–1.484	0.457			
Tumor size						
< 40 mm	Reference					
40–80 mm	1.290	0.460–3.618	0.629			
> 80 mm	0.542	0.197–1.492	0.236			
Grade						
I–II	Reference					
III–IV	0.361	0.116–1.127	0.079			
Unknown	0.652	0.201–2.116	0.476			
T Stage						
T1	Reference			Reference		
T2	0.409	0.135–1.239	0.114	0.638	0.195–2.093	0.459
T3	0.185	0.070–0.491	0.001	0.329	0.113–0.955	0.041
T4	0.176	0.058–0.537	0.002	0.395	0.113–1.383	0.146
N Stage						
N0	Reference			Reference		
N1	0.261	0.135–0.505	< 0.001	0.396	0.184–0.851	0.018
Brain metastasis						
No	Reference					
Yes	0.599	0.242–1.484	0.268			
Liver metastasis						
No	Reference					
Yes	0.513	0.228–1.155	0.107			
Lung metastasis						
No	Reference			Reference		
Yes	0.298	0.149–0.594	0.001	0.411	0.190–0.887	0.023
Chemotherapy						
No	Reference					
Yes	1.279	0.671–2.438	0.454			
Radiotherapy						
No	Reference					
Yes	1.330	0.705–2.511	0.379			
Insurance						
No	Reference					
Yes	0.403	0.049–3.303	0.397			
Marital status						
No	Reference					
Yes	1.348	0.707–2.572	0.364

Moreover, the results of the comparative-ROC in both the training and internal validation sets revealed that the predictive efficacy of the nomogram was better than any of the single independent risk factors (Figs. 7B and 8B). Furthermore, the results of the calibration curves showed that the predicted values of the nomograms were in good agreement with the actual observed values in both the training and internal validation sets (Figs. 7C and 8C). Additionally, in the results of DCA, the nomogram could provide excellent net clinical benefit (Figs. 7D and 8D).

**Figure 9:**
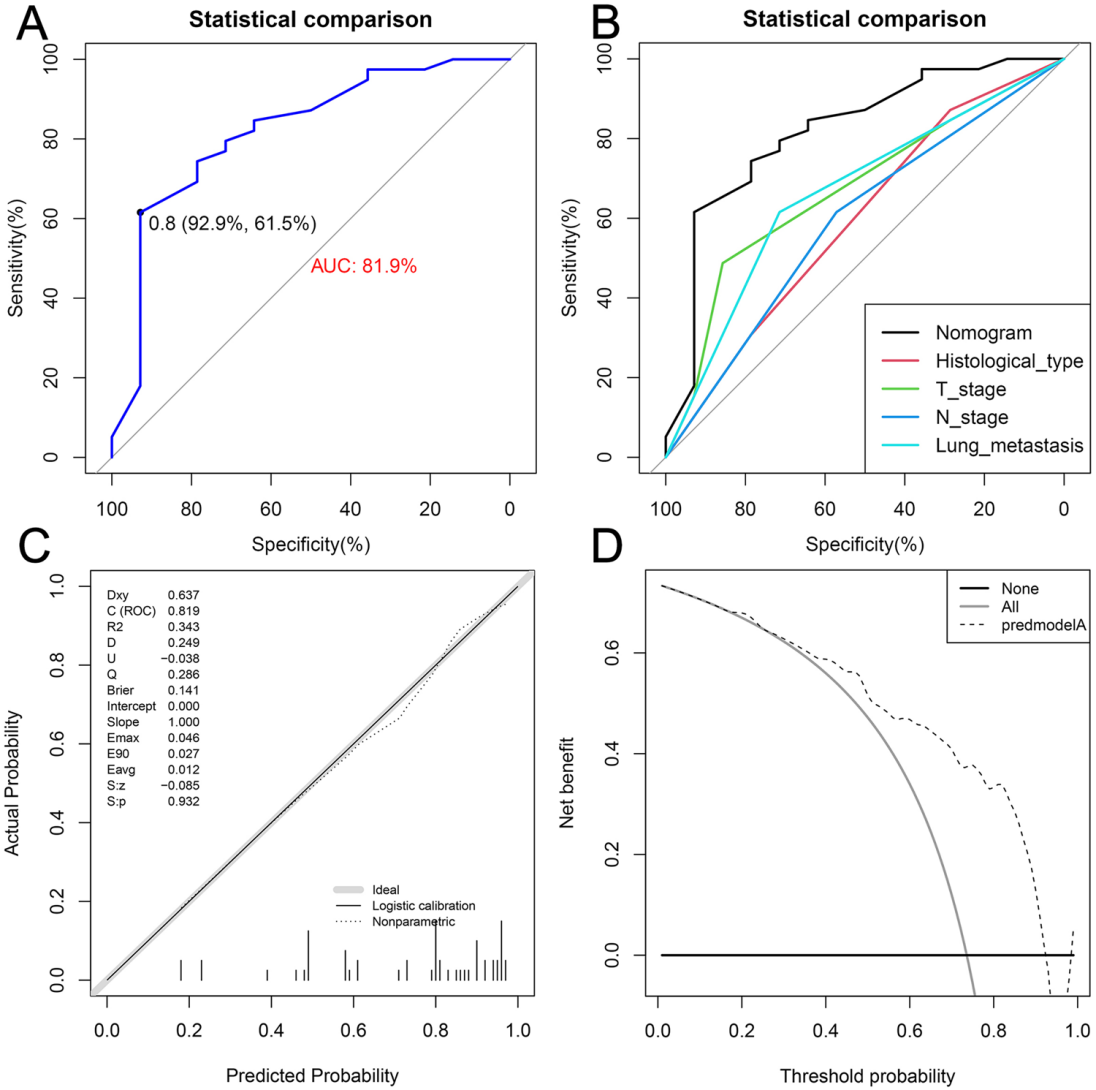
The ROC curve (**A**), comparison of the value of AUC (**B**), calibration curve (**C**) and DCA curve (**D**) of the external validation set. *ROC* receiver operating characteristic curve, *AUC* area under the curve, *DCA* decision curve analyses.

Subsequently, the nomogram also showed excellent validation results in the real external validation set: the AUC of the ROC was 0.819 (95% CI 0.680–0.957), which was better than any other single independent risk factor (Fig. 9A,B). Calibration curve and DCA results again demonstrated the good predictive consistency and clinical utility of the nomogram (Fig. 9C,D).

## Discussion

Numerous studies have demonstrated that resection of the primary tumor considerably enhances survival in KCBM patients^35,36^. As indicated by the study findings, PTR was demonstrated to be a beneficial independent prognostic factor using a substantial cohort from the SEER database (OS: HR = 0.431, 95% CI 0.357–0.478, p < 0.001; CSS: HR = 0.438, 95% CI 0.378–0.507, p < 0.001). Propensity Score Matching is an effective approach for eliminating selection bias and confounding in cohort studies with large samples^37^. Subsequent results following 1:1 PSM consistently demonstrate that PTR serves as a advantageous independent prognostic factors (OS: HR = 0.393, 95% CI 0.325–0.474, p < 0.001; CSS: HR = 0.394, 95% CI 0.325–0.478, p < 0.001). K–M survival analysis similarly confirmed that PTR improves OS and CSS in KCBM patients (Fig. 2). This agrees with a study by Fuchs et al., which similarly demonstrated an improvement in median survival of isolated KCBM from 15 to 36 months following resection of the primary tumor^38^. Similar results were reported by Ruatta et al. Resection of the primary tumor in isolated KCBM could achieve local tumor control and improve survival^39^. However, in some elderly KCBM patients, survival after receiving PTR may be worse than without PTR. This is due to the fact that elderly KC patients are more prone to postoperative complications, which have a high probability of delaying further systemic therapy^21^. Interestingly, many studies support the ability of PTR to provide benefit to patients with bone metastases from kidney cancer, yet others do the opposite. This study suggested that the reason for this phenomenon is due to the lack of accurate screening of patients with KCBM who would not benefit from PTR. The absence of accurate preoperative screening of PTR-beneficial and PTR-Nonbeneficial patients obscures the improved or decreased postoperative overall survival for KCBM patients. As an invasive surgical procedure, the inherent surgical trauma and postoperative complications of PTR have significantly impacted the overall survival of KCBM patients, especially those in poor physical condition^21^. Therefore, nomograms were developed to predict the probability of PTR benefit, which aids in identifying the most suitable surgical candidates for KCBM patients. Adequate internal and external validation has demonstrated the robust predictive capability and clinical usefulness of nomograms to aid clinicians and patients in making informed clinical decisions.

The study confirmed histological type, T/N stage, and lung metastasis as independent risk factors associated with PTR benefits. Based on the independent risk factors outlined above, a nomogram was constructed to quantify the probability of PTR benefits in KCBM patients. Among them, histologic type was most associated with PTR benefits. The prognosis of KC varies according to the histological type, as demonstrated in previous studies^40^. Specifically, PTR contributes to improving the prognosis of both metastatic renal clear cell carcinoma and metastatic renal cell carcinoma^41,42^. And renal cell carcinoma is more likely to benefit from PTR. This might be attributed to the observation that renal cell carcinoma exhibits a lower rate of recurrence^43^. Additionally, lung metastasis has also been shown to strongly correlate with the PTR benefits for KCBM patients. Many studies have reported that isolated BM in KC have better prognosis than multiorgan metastasis^44^. Once lung metastasis occurs in advanced KC, it is frequently accompanied by swift disease progression^45,46^. Due to the lung's rich vascular system, tumor cells are susceptible to involvement in the inferior vena cava and dissemination through the bloodstream^47^. PTR at this time is no longer able to provide the patient with long-term stable survival^48^. This may be due to the fact that tumor cells already have significant impacts on the host's systemic immune regulation, but these mechanisms are not entirely comprehended^49^. Another possible explanation is that multi-organ metastasis adds complexity to the body’s microenvironment, making tumor cells that colonize other sites more harmful and thus reducing the overall benefits of treatment^50^. T stage and N stage have also been shown to be independent risk factors associated with PTR benefits in KCBM patients, with higher staging grades associated with a lower probability of surgical benefits^51^. It is readily understood that higher T stage represents longer period of cancer progression, deeper infiltration of the primary tumor and larger tumor size. This means that the surgeon's surgical program for the patient may be more complex, more surgically invasive, and require a lengthier postoperative recovery process. BM from primary KC is often accompanied by lymphatic system metastases of tumor cells. Out of the 1963 KCBM patients in this study, 693 (35.3%) had metastases in the lymphatic system at the time of initial diagnosis. Higher N stage implies more sites and number of lymph nodes invaded by the primary tumor, which undoubtedly further compromises the survival prognosis of KCBM patients^52^.

The nomogram demonstrated exceptional predictive performance and clinical utility. Furthermore, the extended applicability of the nomogram was well demonstrated in the results of an external validation cohort from another region. Additionally, a web-based nomogram has the potential to facilitate the implementation process into clinical settings^53^. Notably, this study found that patients who benefited from PTR had a median postsurgical OS that was over three times longer than those who did not. Moreover, the overall survival of patients who did not benefit from PTR was not significantly different from that of patients who did not receive PTR. If effective screening of optimal surgical candidates for PTR is not performed prior to PTR, the surgical trauma and financial burden of patients who could not benefit from PTR would undoubtedly be severe. Therefore, it is essential to screen KCBM patients thoroughly for optimal surgical candidacy preoperatively. We suggest that clinicians employ the nomogram derived from this study for precise screening of optimal surgical candidates and optimal decision making in their actual clinical practice.

## Limitations

At this stage, it cannot be denied that this study still has certain limitations. Although the SEER database documented the patient's treatment measures, obtaining more detailed information about the treatment and its implementation timing proved insufficient. Furthermore, the database lacks information on patients' laboratory results and comorbidities. The association between laboratory results and patients' underlying comorbidities and the probability of PTR benefit could not be confirmed. Finally, the nomogram constructed in this study, although validated in a different regional cohort of patients, remains a retrospective analysis. Therefore, further validation by prospective studies is still necessary to verify the results of this study.

## Conclusions

This study indicates that primary tumor resection can offer advantageous survival benefits for KCBM patients. Furthermore, the constructed nomogram quantifies the probability of PTR benefit and allows for accurate screening of optimal surgical candidates.

### Supplementary Information

Supplementary Figures.

## Data Availability

The data for this study were obtained from publicly available databases (https://seer.cancer.gov/). The original contributions present in this study can be found in the article/supplementary material or can be directed to the corresponding author for appropriate reasons.
