# Supplementary material for: A dynamic visualization clinical tool constructed and validated based on the SEER database for screening the optimal surgical candidates for bone metastasis in primary kidney cancer
Source: Sci Rep. 2024 Feb 12;14:3561. doi: 10.1038/s41598-024-54085-x (PMC10861469; doi:10.1038/s41598-024-54085-x)
Supplement: Supplementary file 1 — Supplementary Figures. [file 41598_2024_54085_MOESM1_ESM.docx]

**Supplementary Figure 1.** The standardized mean difference of patients' baseline variables in the PTR and non-PTR groups before and after PSM. PTR, primary tumor resection; PSM, propensity Score Matching.

**
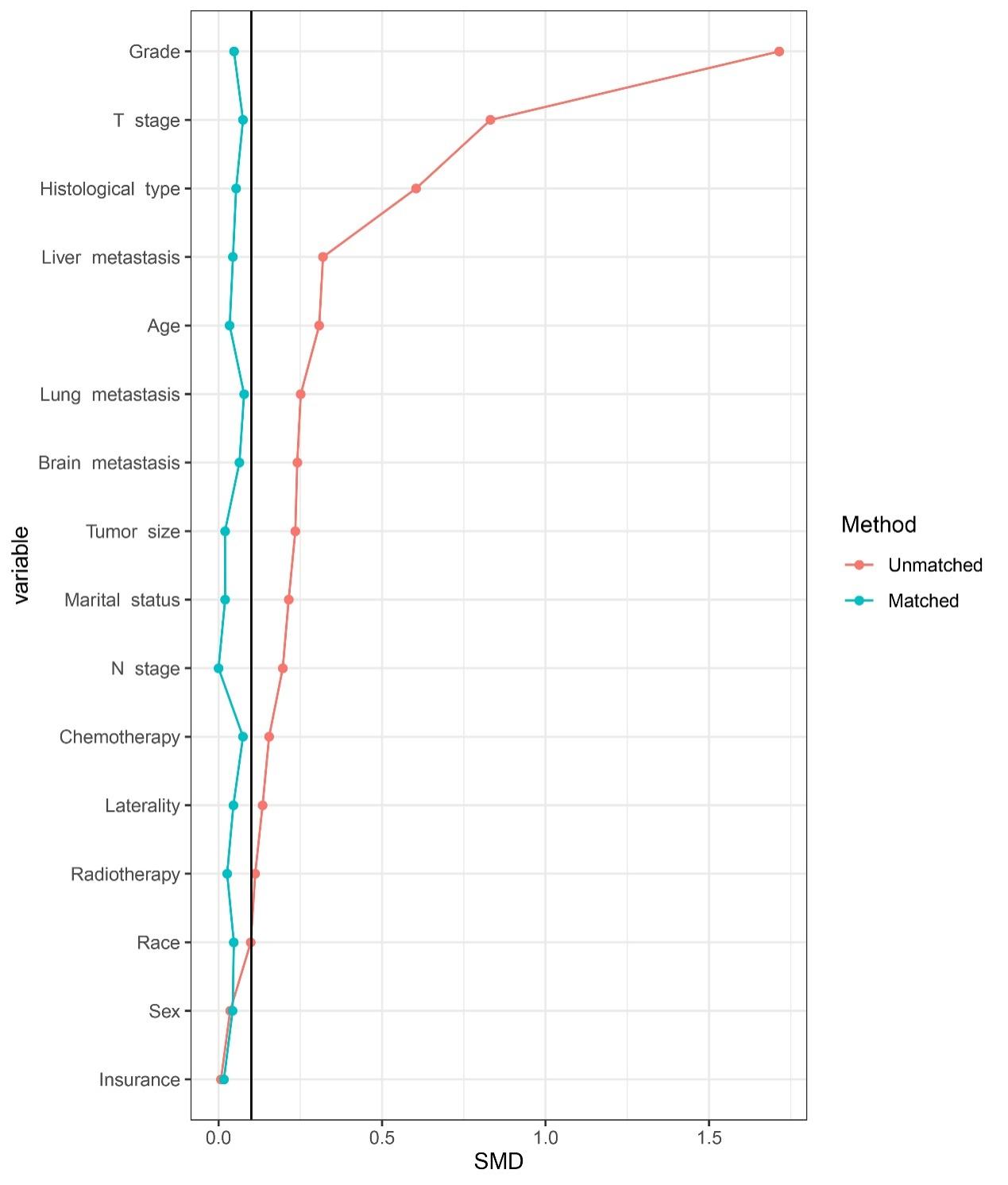
**

**Supplementary Figure 2.** The forest plots for illustrating the results of multivariate Cox regression analysis of OS before PSM (A) and CSS before PSM (B) in KCBM patients. KCBM, kidney cancer patients with bone metastasis; HR, hazard ratio; OS, overall survival; CSS, cancer specific survival; PSM, propensity Score Matching.

**
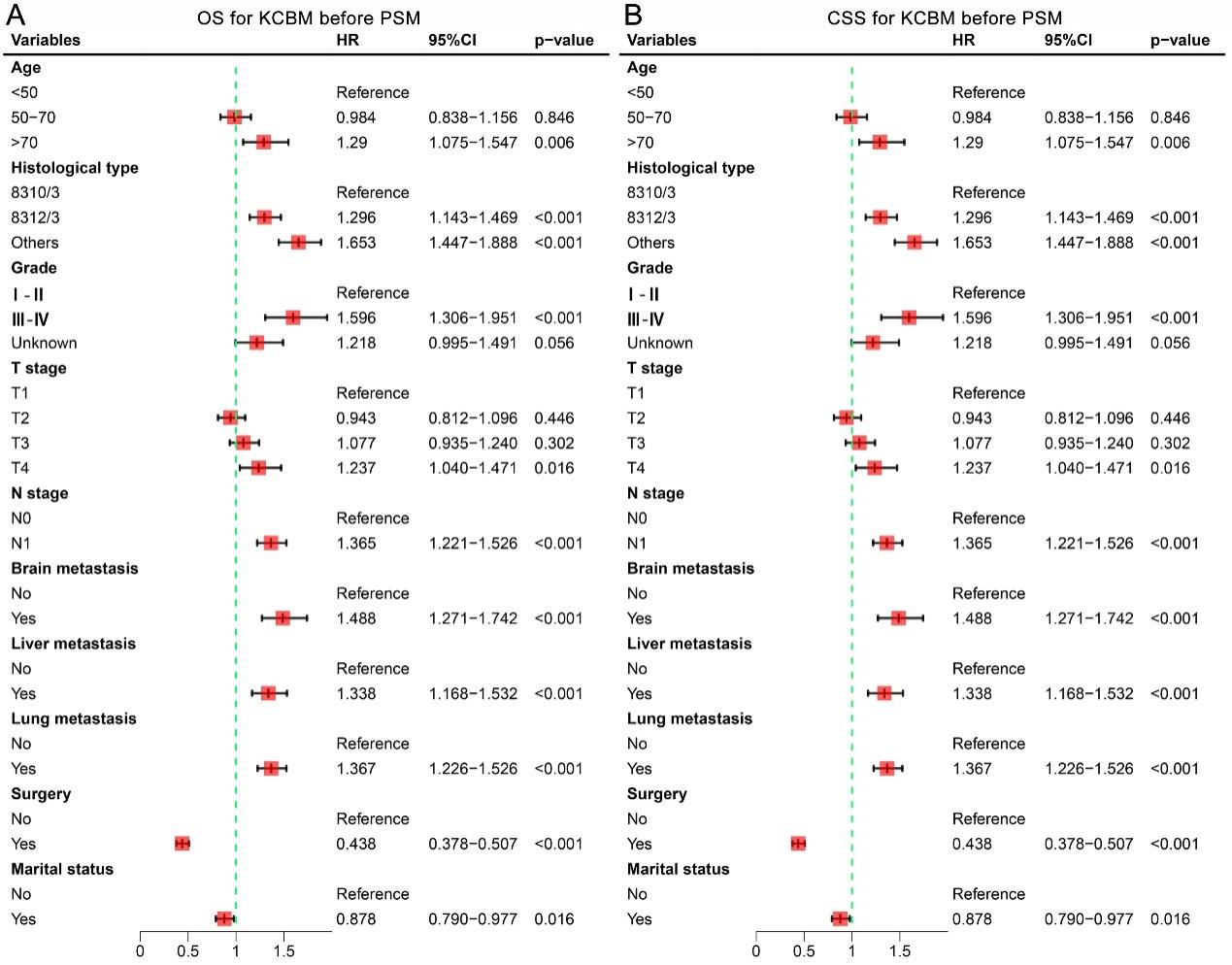
**
